# Thoughts of creation and the discipline of nursing

**DOI:** 10.1002/nop2.239

**Published:** 2019-02-06

**Authors:** Margareth Kristoffersen

**Affiliations:** ^1^ Department of Care and Ethics, Faculty of Health Sciences University of Stavanger Stavanger Norway

## Abstract

**Aim:**

The aim is to highlight thoughts of creation as a significant fundamental of the nursing discipline. This is achieved by exploring thoughts of creation in relation to everyday nursing care.

**Design:**

This study, based on a hermeneutical approach, provides reused data drawn from a larger Norwegian empirical study.

**Method:**

A second thematic analysis was conducted. Data in the original study consisted of qualitative interviews and qualitative follow‐up interviews with 13 nurses. The research context was the primary and secondary somatic and psychiatric health service, inside as well as outside institutions.

**Results:**

Three themes emerged: (a) Life as greater than a human being; (b) creational powers attributed to the human being; and (c) understanding life as basically good. Thus, thoughts of creation in terms of philosophical underpinnings seem to gain backing from nurses’ experiences of everyday nursing care and it can be argued that it adds elements that enrich nursing care.

## 1 INTRODUCTION

This study's point of departure is an interest in highlighting thoughts of creation as a significant fundamental of the nursing discipline by exploring the philosophy in relation to experiences of everyday nursing care. The discipline has as a goal to be of relevance to nurses, implying that nursing knowledge is seen as useful in understanding human health processes and facilitating care for the patient (Griffin, 1980; Risjord, 2010).

Philosophical inquiry has been one important contributor to understanding fundamentals or philosophical premises underlying the nursing discipline and the pursuit of excellence in nursing care (Meleis, 2018; Risjord, 2010). Until the late 1950s, use of the term “nursing discipline” was rare, but from that time onwards, describing nursing knowledge as science‐based emerged in nursing literature (Meleis, 2018; Risjord, 2010). Particularly since the 1980s, different positions have been put forward and debates still revolve around questions such as what constitutes nursing as a discipline (Alligood, 2014a, 2014b). These questions are influenced by political, societal and socio‐economic factors in general (Yeo, 2014) and by philosophy of science in particular (Bluhm, 2014; Meleis, 2018; Risjord, 2010). It has been stated that nursing knowledge must be a solidly argued set of philosophical statements (Butcher, 2004; Risjord, 2010; Uys & Smit, 1994).

At least in the Western countries, nursing knowledge has been influenced by thoughts of creation from its beginning in the middle of the 19th century (Alvsvåg, 2000; Birkelund, 2001; Martinsen, 1984, 2001; Sydnes, 2001) and the philosophy has provided pertinent contributions to the nursing discipline (Delmar, 2006, 2012; Geary & Cone, 2012; Levy‐Malmberg, Eriksson, & Lindholm, 2008; Martinsen, 1996; Wolf & Bailey, 2013). The contributions are pertinent particularly because thoughts of creation address fundamental questions revolving around life, that is what is life and a human being?, what is of value or of importance in life?, and what is good? (Alvsvåg, 2000; Pesut, 2008). Various life phenomena such as relationality or inter‐dependence, love and trust are emphasized and regarded as given life conditions (Delmar, 2006, 2012; Levy Malmberg et al., 2008; Martinsen, 1996; Thorkildsen, Eriksson, & Råholm, 2013). Human beings’ dignity has also been connected to a given position in life, originating from thoughts of creation and described as an indestructible human value (Edlund, Lindwall, von Post, & Lindström, 2013).

Considering various life phenomena as given incorporates that they are related to life and not solely to human beings or are of human beings’ own making (Hansen, 1996, 1999). Thoughts of creation thereby offer a complementary perspective on other philosophical perspectives linked to the nursing discipline, for example, virtue ethics (Sellman, 2000, 2011). Virtue ethics emphasizes the nurse's internal goods (Tyreman, 2011), such as personal values, skills and knowledge, or the moral character of the nurse, as being integral to and defining nursing care (Newham, 2015). Such a perspective interprets life in a restricted manner by underestimating the fact that life can be seen as created. It represents a view which will never be fully supported, as creational powers in life also influence the created world (Hansen, 1996, 1999).

As described in this study, thoughts of creation represent universal human or philosophical perspectives having their origin in very old oriental oral traditions (Hansen, 1996, 1999; Løgstrup, 1995, 1997; Martinsen, 2012) and do not primarily represent a Christian or theological idea of creation. The philosophy has influenced culture, society and community for decades and basically represents human and universal statements about thoughts of creation which are possible to relate to each human being (Henriksen, 2014). However, at least in the Western countries, thoughts of creation are best known as they are written in the Bible, more precisely, as an account of creation in Genesis in The Old Testament (Geary & Cone, 2012; Hansen, 1999). Pointing out the significance of standing against such a background is in line with Tyreman (2011), who has emphasized the importance of bringing into focus external goods, such as culture, society and community as a background nurses are representing and from which they gain their identity.

It may then be important to highlight thoughts of creation in the nursing discipline by exploring them in conjunction with experiences of everyday nursing care. There seems to be little documentation of how the philosophy can be linked to such experiences despite nursing literature having thoughts of creation connected to the discipline. This indicates a need for more in‐depth understanding of what is claimed to be a shortcoming. An argument for this is that beneath the surface, nurses’ everyday experiences may be linked to aspects in the philosophy, even though these are rarely discussed. Central aspects of the philosophy can support and advance everyday nursing care. Bringing thoughts of creation into focus by drawing on experiences from nursing practice may be one way of vitalizing the philosophy as a fundamental of the nursing discipline. Nevertheless, it is through what can be said about the philosophy that such an understanding is provided. This study aims to highlight thoughts of creation as a significant fundamental of the nursing discipline.

## 2 BACKGROUND

Although there is no clarity regarding what the term “creation” exactly comprises, in this study it is described as based on a thought‐motive called “primeval‐history” (Hansen, 1999). The thought‐motive incorporates that creation can be understood as a universal phenomenon and common to all mankind (Hansen, 1999; Løgstrup, 1995). Creation is consequently a phenomenon that goes on in the created world and that life itself tells us about (Hansen, 1999). This means that creation goes on in life itself, here and now and at any given moment we are alive and live. Another way of stating this is to say that the thought‐motive of creation does not portray the created world as something that was created once upon a time in the distant past—the created world is not created once and for all. Thus, the thought‐motive makes it possible to consider creation as an understandable phenomenon (Hansen, 1999).

Here, it is worth noting that creation and describing life as “created” does not mean excluding the fact that all the created has a standing in itself (Henriksen, 2014, p. 109). The thought‐motive of creation implies that the human being has the capacity to be a co‐creator (Hansen, 1996; Henriksen, 2014). Henriksen underlines the importance of this when he says that the human being for the most part is not immediately given, but its shape and character are socially constructed (2014, p. 43). This involves the human being as not being seen as created once, as creating involves constantly doing something new to meet what the human being needs (Hansen, 1996; Henriksen, 2014).

Emphasizing the assumption that creation is often characterized by the term “gift” means that life is understood as “given” (Hansen, 1996, 1999). Løgstrup (1995) states that human beings by themselves have never caused their own life; on the contrary, life has been given to us and is an ongoing gift. Life is essentially given to us as something good, implying that the created world gives rise to a pleasure simply because it is created and alive. Although life can be seen as a precious gift and thus, not as a subordinate good, it is important to point out that the thought of creation does not exclude the fact that life can change and become bad when boundlessness occurs in life or humanity (Hansen, 1996; Henriksen, 2014; Løgstrup, 1997). Boundlessness can be described as taking responsibility to the point of having no limits and in the worst case, leads to encroachment (Løgstrup, 1997). This incorporates that thoughts of creation do not include considering everything in life as equal.

Furthermore, thoughts of creation open up for an understanding of life as being given by a creational power, which is possible to describe as creator of the world, meaning a power in and beyond human beings’ experiences (Henriksen, 2014). In this study, creational powers in life are described as a force which is something more than the created, even extending beyond it. Extending beyond involves staying behind the created as creator, implying being hidden or having no empirical reality that is similar to anything in the created world (Henriksen, 2014, p. 20). Such a power can be called a divine or God (Hansen, 1999). A creational power in life can also be described as metaphysical. Løgstrup (1995) claims that this implies widening understanding of the power by closely connecting it to life as such. Foss (2005) clarifies that such an extension involves creational power being understood as life itself; thus, it is more loosely connected to the divine or God. This means that a divine affirmation of the creature can also be more loosely connected to a divine or God.

Extending beyond the created also involves the power being distinct, unlike or different from the created because the created is always visible and alive on earth (Henriksen, 2014). Importantly, this distinction does not exclude a close relation to the created world. Løgstrup (1995) states that such a power influences the created world. It promotes life and diversity in life by creating order and coherence and it maintains and is involved in each moment the created world is alive, implying that the created world is “willed.” Being “willed” means having a central place in the created world, which again gives the human being dignity and integrity (Henriksen, 2014). The power is perceived as a basic condition to keep “nothingness” away from the created and this matches the fact that whatever exists perishes and comes to an end.

## 3 METHOD

### 3.1 Design

This study is a second analysis using a hermeneutical approach (Lindberg, Österberg, & Hörberg, 2016). It is based on a research question concerning data derived from a previous, larger Norwegian empirical study which used a hermeneutical research design (Taylor, 1999) with the aim of interpreting what is of significance for remaining in nursing practice (Kristoffersen, 2013). Data in that empirical study inspired to carry out a second analysis, with the intention of exploring the significance of thoughts of creation in relation to how nurses expressed experiences of everyday nursing care. It was a more in‐depth understanding of the empirical data in light of thoughts of creation that had emerged as a matter of interest (Heaton, 2004) and using that philosophy to understand the data further was considered as valuable (Lindberg et al., 2016).

### 3.2 Sample and participants

The sample in the larger original study was based on non‐probability method (Kristoffersen, 2013). Participants’ selection criteria were a minimum of 2 years’ nursing experience and full or almost full‐time work. There were 13 participants aged between 26–62 years (median 51 years), with varying work experience in the primary and secondary somatic and mental health service, from inside as well as outside institutions. Their work experience ranged from 2–40 years. Many of the participants had worked 10 years or more on the same ward and were in full or almost full‐time employment.

### 3.3 Data collection

The larger original study was conducted with the aid of qualitative interviews and qualitative follow‐up interviews (in all 27 interviews) (Kvale & Brinkman, 2009; Silverman, 2006). Qualitative follow‐up interviews were used to deepen the already collected data about day‐to‐day experiences of caring for patients. In the interviews, the participants were asked to freely describe what is of significance for remaining in nursing practice.

In this second study, data were collected from transcripts of qualitative interviews in the larger original study. The transcripts were read again to identify the parts of the empirical material considered as the most suitable to explore thoughts of creation in relation to how nurses expressed experiences of everyday nursing care, along with an endeavour to comprehensively understand these expressions (Heaton, 2004).

### 3.4 Ethical consideration

The reused data are drawn from an original empirical study approved by the Norwegian Center for Research Data (Kristoffersen, 2013). Information was given, and consent obtained from the participants. The reused data was limited and anonymized, so the participants were not contacted again.

### 3.5 Analysis

Data analysis in the larger original study was based on a phenomenological hermeneutic approach. The different steps in the analysis were narrative reading, different thematic readings and a comprehensive understanding (Lindseth & Norberg, 2004).

The second data analysis was inspired by some methodological support principles (Lindberg et al., 2016). It was performed by reading the reused part of the empirical data to gain a general structure of the material and find new patterns of meanings. In the next step, a philosophical examination was performed whereby the data and the philosophical texts were read again (Lindberg et al., 2016). The reading involved conducting the analysis process of exploration with an open attitude to get an understanding of data in relation to thoughts of creation, implying a process of deep reflection (Lindberg et al., 2016). The analytical strategy was to openly question the material, asking, for example, what the data tells about creation and creational powers in life in relation to experiences of everyday nursing care and how thoughts of creation can shed light on the experiences. Also, in the reused data, it was considered what appeared to be obvious and what appeared to be more latent meaning structures. A more in‐depth understanding of the data emerged (Lindberg et al., 2016), resulting in descriptions of three themes.

### 3.6 Trustworthiness

The trustworthiness of this study is considered a strength (Lincoln & Guba, 1985), as the reused data demonstrates everyday nursing experiences and was considered as relevant in interpreting central aspects of thoughts of creation. This implies that the data can be seen as suitable (Heaton, 2004). It can also be seen as a strength that the original empirical study used phenomenological philosophy as one theoretical perspective (Kristoffersen, 2013). Furthermore, the interpretation of the data has been conducted with openness and reflection (Lindberg et al., 2016). Nevertheless, there is still a risk that an interpretation of experiences of nursing care on a comprehensive level implies an over‐interpretation of the reused data. To delimit that risk, alternative interpretations in light of thoughts of creation were discussed in the research group until consensus was reached. This indicates that the analysis was performed in such a way that the study's findings were found to be credible (Lincoln & Guba, 1985).

It is relevant to point out possible limitations linked to reusing data to explore thoughts of creation in relation to nursing experiences. One could view such an exploration as superfluous because it was a deductively inspired analysis. Another critical aspect is how well the reused data fit the present study's aim (Heaton, 2004). The data may be considered limited because it was translated from Norwegian to English and thereby loses some of the naturally occurring richness in daily language.

## 4 FINDINGS

By exploring thoughts of creation in relation to how nurses expressed experiences of everyday nursing care, three themes emerged: (a) Life as greater than a human being; (b) creational powers attributed to the human being; and (c) understanding life as basically good.

### 4.1 Life as greater than a human being

The data shows that the nurses used words such as “something divine, a spirituality, something unifying in life” (1) or “‘the one’ up there” (2) when they talked about life related to everyday nursing care. Even though these words were not connected to a specific religion, they can be understood as a description of creational powers in life and more precisely, as articulations of phenomena in life that cannot be verified in the same way as the created world. It is therefore interesting to hear how one nurse went on to explain:

Even though I can’t always understand life, I believe there is a meaning to life. I try to believe that the meaning of life must be good, both for myself and others. There must be something good and meaningful in this life and not solely coincidences and meaninglessness. I believe there is a God behind life and in life and that I’m quite small and understand very little of it. (3)

The expression “I believe there is a God behind life and in life” illustrates how creational powers were described as phenomena that were experienced as something greater than the human being, implying going beyond and staying behind life. This is also evident from the nurse's comment: “I believe there is a meaning to life.” Interpreting creational powers as something greater than the human being involves there being a difference in relation to the created world. This difference may be connected to a capability to create life and keep nothingness from life. Being in a relationship to such powers can provide a feeling of being in a close relation and not standing alone in nursing care. It seems to contribute to what makes life worth living and strengthens the hope that life will be good. This could be important when life is experienced as difficult to understand, particularly in terms of the patients and their life conditions. Comments such as “I can't always understand life” and “I'm quite small and understand very little of it” can be seen as an expression of how the nurse experienced not having absolute control in life, that is that life was not experienced as anthropogenic and thus, it was not solely the responsibility of nurses to decide what should live or die. This means that life presents itself in everyday nursing care in ways that the nurses were not directly able to change. In turn, neither nurses nor patients have full control in everyday nursing care. By saying “I try to believe” the nurse demonstrated her openness to uncertainty related to incidents in life, although at the same time, striving to achieve control related to nursing care seemed to be important. Another nurse said more concretely that: "*I can do my best, but then I can't do more as we are only human beings*"(4).

### 4.2 Creational powers attributed to the human being

The data demonstrates how the essence of the patient cared for in nursing care was understood. One nurse said:

A creational power in the human being separates us from other living beings, meaning we all have creational powers. It is a power which, as a core, is possible to extract from ourselves and also poke out of others. The most important prerequisite to be able to work with patients may be that all human beings have such a power in themselves. (5)

Here, it is possible to discern how the nurse attributed creational powers to the human being. The nurse described “creational power” as something “in” the human being, implying that the powers are understood as an embodied quality. This quality “separates” human beings from “other living beings.” Considering creational powers in the human being can be understood as the way the nurse recognized the patient's dignity and integrity, meaning that the patient has human value.

Further, a prerequisite for the nurse's work with patients may be confidence in life's renewal processes. Extracting “a core from ourselves” and “poke” it “out of others” can be seen as expressing how the nurse articulated confidence in life by having an openness to the new, that is that which is different to now. Pointing this out is possible because creation is described as essentially related to a future that may imply change and an expansion of present life conditions. In other words, life's renewal processes might be helpful, particularly because they can bring a sense of something new to be created in the patient's life conditions—something new that may also be good and thus, worth “poking out.” Additionally, the nurse's comment demonstrates how she wanted to contribute to this kind of well‐being. This interpretation involves considering the nurse as co‐creator of a continued creation. Thus, extracting creational powers out of oneself as a nurse and poking such a core out of the patient can be understood as an articulation of how the nurse realized being a co‐creator in everyday nursing care, implying that the nurse has such a capability. Put otherwise, confidence in life's renewal processes can require of the nurse to “ride on the bright moments” by rousing the patient's spark of enthusiasm (6).

### 4.3 Understanding life as basically good

The data demonstrates that understanding life as basically good does not mean ruling out the bad. One nurse said:

I have expectations of life, like “that’s life” and so when bad things happen I can confront and endure it. Experiencing bad things does not imply a disaster—it hurts and is uncomfortable—but that’s life right now and you get through that phase. We will encounter good things again, or life calms down. That’s how life is: good and bad. (7)

This quote can be understood as an expression of how the nurse integrated different nursing experiences which appear as contrasts to each other in a whole. Here, one could point out that describing life as both “good and bad” demonstrates the nurse did not see the good as completely incompatible with an understanding of life as less good or bad. More concretely, having “expectations of life, like ‘that's life’” contributed to confronting life as it is. An experience of how everything in life belongs to life and consequently that the good and the bad in life do not stand apart seemed to stimulate the nurse's capacity to meet life as it really is “right now,” implying helping the patient when “bad things” in life hurt to “get through that phase.” In other words, it can be perceived as an expression of how life essentially understood as “given” inspired the nurse simply because life is created and will give “good things again”. Another nurse said the following about how understanding life as basically good inspired everyday nursing care:

The bad things of today is possible to solve because we have managed things like this before. When our hard work promotes life and the patient gets a better day, it gives a pleasure as there has been a movement from the bad to the good. (8)

Additionally, the data demonstrate that proximity to patients and their life conditions means nurses are particularly exposed to all facets of life. One nurse stated:

Life itself is a challenge and it’s pretty tough. But we will be more whole human beings if we live the life we have been given and meet the challenges we get. (3)

Here, it is possible to see how the nurse's experiences of bad things in life did not necessarily include wishing that such things had never happened or in the worst case, seeing them as a curse. An experience described as being “more whole human beings” seems to emerge because life understood as “good” stands its ground. This means that the nurse's experiences of the good in life can be understood as one contributor to remaining in life and moving forward, implying that bad experiences, at least over time, can be changed to something less bad. The nurse nevertheless pointed out a prerequisite for such a change: to “live the life we have been given.” Importantly, the data can also be perceived as an expression of how the nurse understood the human being as a subject in one's own life, meaning that the good and bad in life also depend on psychological or emotional aspects. Emphasizing this related to nursing care might imply having access to these inner realms to promote being a subject in one's own life, either as a nurse or a patient.

## 5 DISCUSSION

The study's overall aim was to highlight thoughts of creation as a significant fundamental of the nursing discipline by exploring them in relation to experiences of everyday nursing care.

The findings have demonstrated how the nurses experienced life as greater than a human being. They talked about creational powers as being greater than a human being, meaning that life presents itself in everyday nursing care in ways that the nurses were not directly able to change, that is in terms of the patients and their life conditions. Explicating an insight that acknowledges that, as human beings, we do not have full control related to life and death can contribute to clarifying the limitation of human beings’ power in relation to life, as powers exist which can be understood as extending beyond the human (Hansen, 1996; Løgstrup, 1995). This means that nurses cannot prevail over life itself, as life is greater than a human quality; the world is created and whatsoever exists perishes and ends. Such an insight can illuminate everyday nursing experiences, particularly when nursing care requires recognizing nurses’ experiences of how they, as professionals, can be limited in relation to patients’ nursing care. Although limitations of human powers exist in relation to how life presents itself, the insight does not exclude that a nurse can help a patient and that relieving suffering makes a difference (Thorkildsen et al., 2013). It is emphasized that nursing care should be considered as related to serving life and helping the patient to live (Kristoffersen & Friberg, 2015).

The study's findings have also demonstrated how the nurses attributed creational powers to the human being, thus emphasizing the worth of the patient cared for. Thoughts of creation contribute to focusing on the dignity of a human being, as therein lies divine affirmation of the creature (Hansen, 1996; Henriksen, 2014). The philosophy highlights that the human being is created and consequently given as something good, understood as one embodied quality and the human being can thus be received as something worthy. This is also underlined by Edlund et al. (2013), who describe human worth as an absolute worth, meaning a lasting and inviolable dignity which originates from thoughts of creation. It is nonetheless important to state that the human being has a standing in itself and a capacity to be a co‐creator in life, implying that the view does not stipulate that human beings are unable to determine and shape their lives in relation to the future (Hansen, 1996; Henriksen, 2014). On the contrary, it contributes to focusing on opportunities in life. Another way of stating this is to say that receiving life as a “gift” has an impact: giving something in return for what is given by taking care of the created world, that is the patient and thereby contributing to a continued creation of life (Henriksen, 2014).

It was an important finding how nurses used their capacity to be a co‐creator. By extracting creational powers out of oneself as a nurse and poking such a core out of the patient, the nurses thereby contributed to realizing their lives’ renewal processes and promoting the patients’ dignity. This involves nurses saying a new “word” or giving a new “message” to the patient when needed**—**a finding in line with previous research. Edlund et al. (2013) have pointed out that human beings have been given a unique position in the created world and have an obligation to serve life, meaning a freedom given to them by creation and thus, a choice regarding how to relate to a situation. It has been documented that preserving the patients’ dignity revolves more concretely around allocating time to the patient, inviting the patient to participate and shielding the patient's body (Valeberg, Liodden, Grimsmo, & Lindwall, 2018). Previous research has also documented that psychological or emotional aspects are interwoven in the nurse's actions and decisions (Nortvedt, 2014). The nurses’ character might then motivate them to realize nursing care for the patient's best (Newham, 2015) and values such as beneficence inspire nurses to promote patients’ well‐being and avoid harm to patients (Holm, 2001; Newham, 2015; Nortvedt, Hem, & Skirbekk, 2011; Sellman, 2000, 2011).

Moreover, the study's findings indicate how everyday nursing care was connected to an understanding of life as basically good. The good and the bad were nonetheless intertwined related to everyday nursing experiences. A similar observation is also demonstrated in a previous study (Kristoffersen, Friberg, & Brinchmann, 2016). By taking into consideration that everything in life is a part of life, thoughts of creation provides a view of life understood as both good and bad, implying that the bad is necessary in life, although never so much as the good (Hansen, 1996; Henriksen, 2014; Løgstrup, 1995, 1997). Rather, the good and the bad are seen as contrasts and mutually dependent on each other, despite being qualitatively different. The point here is that the bad is necessary in life, but nonetheless never so much as the good (Henriksen, 2014). The nurses’ experiences can therefore be used to support the significance of including an openness to life, where everything in life is not understood as equal. Adding insight into how the good and the bad in life are intertwined and do not stand apart can strengthens nurses’ capability to meet all facets of the patient's life as it is experienced in nursing care.

### 5.1 Implications

It is relevant to consider some issues connected to thoughts of creation in relation to the nursing discipline as the study's findings point to thoughts of creation as significant nursing knowledge. As a fundamental, the philosophy becomes a valuable tool, providing a human and universal perspective to the nursing discipline. This can be argued as central aspects of the philosophy seem to be manifested in nursing practice. Thoughts of creation in terms of philosophical underpinnings have gained backing from nurses’ experiences of everyday nursing care and such experiences can be important simply because they are expressed by nurses as nursing experiences and appear to nurses as part of life. This indicates that the philosophy which has influenced culture, society and community for decades (Hansen, 1996, 1999; Henriksen, 2014) might also be useful to nurses in understanding human health processes and facilitating care for the patient.

More specifically, the philosophy is a valuable tool, providing a supplementary view of life to other philosophical perspectives linked to the nursing discipline as these perspectives interpret life in a restricted manner by underestimating the fact that life can be seen as created (Newham, 2015; Sellman, 2000, 2011; Tyreman, 2011). The philosophy is a supplementary tool, adding elements that enrich the nursing discipline. It provides avenues for thinking differently largely because central aspects of the philosophy add insight on how life presents itself as greater than human beings, creational powers is attributed to the human being and life is understood as basically good (Hansen, 1996, 1999; Henriksen, 2014). Previous nursing research points to the importance of standing against a background which addresses questions revolving around what is life and a human being (Alvsvåg, 2000; Delmar, 2006, 2012; Levy Malmberg et al., 2008; Martinsen, 1996; Pesut, 2008).

On the other side, when considered as an ideology, the philosophy can be understood as evolving into a kind of fixed structure. It can be argued that this may generally be problematic in that it involves understanding life as narrow, a notion that might be used as an infringement or, in the worst case, a reason for abuse (Løgstrup, 1997). This implies a risk of switching to an ideology which determines what is right or wrong ethos (Hussey, 2009). Linked to everyday nursing care, such a switch could happen if the practical reality of nursing demands nurses to adhere, accept and favour thoughts of creation as a condition of being a nurse or of delivering high‐quality nursing care. This may, however, be an impossible scenario to support. Instead, it is important to emphasize that the philosophy is disputed and can be criticized for not having concepts with a clear definition (Hansen, 1996; Løgstrup, 1995). Delineating what counts as such thoughts are dependent on context. Consequently, they can be weakened by other philosophical perspectives such as core virtues (Sellman, 2000, 2011), ethical guidelines and principle‐based ethical theory (Nortvedt, 2001, 2014). The nursing discipline should therefore not neglect to base nursing care on ethical guidelines and principle‐based ethical theory and values, such as non‐maleficence. Nonetheless, there is a distinction between seeing nursing as dependent on thoughts of creation and seeing the philosophy as complementary to other perspectives influencing the nursing discipline. As a supplementary tool, thoughts of creation can offer central aspects and thereby enrich nursing knowledge, even though these aspects are not concepts with a clear definition (Swinton & Pattison, 2010).

Additionally, thoughts of creation have religious undertones, which might make it difficult to gain significant popularity in nursing care. It is not unproblematic to introduce the philosophy, implying that it can create difficulties which can be hard to overcome. It is nevertheless important to do so, for the following reasons. Firstly, it introduces a view whereby life is not solely understood as anthropogenic or having its origin there, meaning that life is not given by human beings themselves (Hansen, 1996, 1999). Another argument is that connecting thoughts of creation more loosely to a divinity or God allows religious and non‐religious perspectives to be equated. These perspectives have at all times mutually influenced each other (Løgstrup, 1997), implying that each life phenomenon and context that is open for a religious interpretation can also be interpreted as non‐religious. It can be argued that framing in this way is relevant to the fact that Western countries have more or less lost a religious perspective on life, while a more non‐religious perspective has surfaced (Henriksen, 2014). Moreover, taking into consideration that there might exist great diversity among religious ideas today, it is important to emphasize that thoughts of creation are human and universal statements moving beyond distinctions between religion, philosophy and science (Hansen, 1999; Hussey, 2009; Paley, 2008).

## 6 CONCLUSION

Highlighting thoughts of creation as evident in the nursing discipline may be of relevance in illuminating how life presents itself in everyday nursing care and what it basically revolves around, that is how creation goes on in life here and now, implying that it is important to be present at any single moment in caring for the patient. Although the nurse's power related to life and death can be experienced as limited, he or she has a capacity to be a co‐creator of a continued creation, where the good and bad can be intertwined in everyday nursing care. Additionally, thoughts of creation provide a language which can bring nurses’ experiences into perspective, giving them depth and nuance. The added value could be that the philosophy provides a vehicle for describing aspects that nurses might find most difficult to be aware of and grasp last of all. This raises further questions of how thoughts of creation can enrich everyday nursing care and contribute to future development of nursing knowledge.

## CONFLICT OF INTEREST

The author declare that there are no conflicts of interest with regard to this study.
